# Bacterial etiology of bloodstream infections and antimicrobial resistance in Dhaka, Bangladesh, 2005–2014

**DOI:** 10.1186/s13756-016-0162-z

**Published:** 2017-01-05

**Authors:** Dilruba Ahmed, Md Ausrafuggaman Nahid, Abdullah Bashar Sami, Farhana Halim, Nasrin Akter, Tuhin Sadique, Md Sohel Rana, Md Shahriar Bin Elahi, Md Mahbubur Rahman

**Affiliations:** International Centre for Diarrhoeal Disease Research, Bangladesh (icddr,b), Dhaka, 1212 Bangladesh

**Keywords:** Bloodstream infection, BSI, Antimicrobial resistance, Multidrug-resistance (MDR), Epidemiology, Gram-positive bacteria, Gram-negative bacteria, Dhaka, Bangladesh

## Abstract

**Background:**

Bloodstream infections due to bacterial pathogens are a major cause of morbidity and mortality in Bangladesh and other developing countries. In these countries, most patients are treated empirically based on their clinical symptoms. Therefore, up to date etiological data for major pathogens causing bloodstream infections may play a positive role in better healthcare management. The aim of this study was to identify the bacterial pathogens causing major bloodstream infections in Dhaka, Bangladesh and determine their antibiotic susceptibility pattern.

**Methods:**

From January 2005 to December 2014, a total of 103,679 single bottle blood samples were collected from both hospitalized and domiciliary patients attending Dhaka hospital, icddrb, Bangladesh All the blood samples were processed for culture using a BACT/Alert blood culture machine. Further identification of bacterial pathogens and their antimicrobial susceptibility test were performed using standard microbiological procedures.

**Results:**

Overall, 13.6% of the cultured blood samples were positive and Gram-negative (72.1%) bacteria were predominant throughout the study period. *Salmonella* Typhi was the most frequently isolated organism (36.9% of samples) in this study and a high percentage of those strains were multidrug-resistant (MDR). However, a decreasing trend in the *S*. Typhi isolation rate was observed and, noticeably, the percentage of MDR *S. Typhi* isolated declined sharply over the study period. An overall increase in the presence of Gram-positive bacteria was observed, but most significantly we observed the percentage of MDR Gram-positive bacteria to double over the study period. Overall, Gram positive bacteria were more resistant to most of the commonly used antibiotics than Gram-negative bacteria, but the MDR level was high in both groups.

**Conclusions:**

This study identified the major bacterial pathogens involved with BSI in Dhaka, Bangladesh and also revealed their antibiotic susceptibility patterns. We expect our findings to help healthcare professionals to make informed decisions and provide better care for their patients. Also, we hope this study will assist researchers and policy makers to prioritize their research options to face the future challenges of infectious diseases.

**Electronic supplementary material:**

The online version of this article (doi:10.1186/s13756-016-0162-z) contains supplementary material, which is available to authorized users.

## Background

Bloodstream infection (BSI) due to bacterial pathogens is a global concern. It is often associated with increased length of hospital stay, a significant amount of healthcare related costs and most significantly, a high rate of morbidity and mortality [1]. Depending on the age, severity of infection and other risk factors, the mortality rate for BSI varies between 4.0 and 41.5% [2–7]. Recent studies have reported a rapid increase in the number of bloodstream infections from both community and nosocomial sources [8, 9]. *Staphylococcus aureus*, *Streptococcus pneumoniae* and *Escherichia coli* have been found to be the most commonly isolated pathogens associated with BSI worldwide [3, 6].

The epidemiology of BSI varies depending on the geographic location, age and co-morbid illnesses. As an example, *Salmonella enterica* is a frequently isolated pathogen from blood samples in both African and Asian regions, however their serotypes differ substantially [10]. *S.* Paratyphi is the predominant organism in the *Salmonella* group in Africa whereas *S.* Typhi is the most frequently isolated organism in Asia. Besides their isolation rate, their antibiotic susceptibility pattern varies substantially [10]. So, understanding of local epidemiology may play an important role in making proper empirical treatment choices before laboratory test results are available. This is especially true for Bangladesh and other developing countries where healthcare systems operate on poor hygiene system and lack proper facilities to contain infections. In these countries, early treatment is usually based on the patient’s clinical symptoms rather than diagnostic results. Therefore, patient’s early prognosis to final outcome might be much improved by available epidemiologic data for the most frequently isolated pathogenic organisms. However, complete data on BSI causing organisms from Dhaka, Bangladesh is scarce.

In this study, we aimed to identify the most prevalent bacterial pathogens involved in BSI in hospital and domiciliary patients from Dhaka, Bangladesh. We also determined pathogen antibiotic susceptibility patterns to evaluate the changing trend of antimicrobial susceptibility in this region.

## Methods

In this retrospective study, blood samples were obtained from patients attending out-patient and in-patient services at Dhaka hospital, icddrb, Bangladesh which is a primary care hospital with 200 in-patient beds. A total of 103679 blood samples were processed from January 2005 to December 2014. In the consecutive ten years of the study 9600, 9364, 9189, 9523, 9523, 11578, 11179, 10822, 10161 and 12740 samples were received and processed from 2005 to 2014 respectively. All the blood samples were processed for culture using a BACT/Alert blood culture machine to identify the presence of bacterial pathogens. Antimicrobial susceptibility tests were performed on the isolated pathogens using standard microbiological procedures [11].

### Bacterial isolation

Collected blood samples were directly inoculated into adult (more than 12 years of age) and pediatric (up to 12 years of age) FAN blood culture bottle. Bottles were incubated in the BACT/Alert machine for up to 5 days. Positive culture samples were directly inoculated onto MacConkey (MC) agar, chocolate agar and blood agar (5% sheep blood) plates. MC plates were then incubated at 35 °C in aerobic condition. Chocolate and blood agar plates were incubated at 35 °C in microaerophilic condition (containing 5% CO_2_). Bacterial pathogens were identified using standard bacteriological procedures [11]. API identification strips (bioMérieux, France) were used as supportive tests for further identification.

### Antimicrobial susceptibility testing

Antimicrobial susceptibility tests were performed by using the disk diffusion method and susceptibility patterns were determined following CLSI guidelines [11–14]. The study was begun following CLSI, 2004 guidelines [14] and later the relevant changes made in 2010, 2012 and 2013 by CLSI were incorporated. Breakpoint changes made in 2010 and 2013 for carbapenems in the case of *Enterobacteriaceae* [12, 13] were adapted*,* and also the revised ciprofloxacin breakpoint for *Salmonella* in 2012 [11]. Antibiotic susceptibility was tested for CN (10 μg), SXT (25 μg), Cip (5 μg), CRO (30 μg), AMP (10 μg), Caz (30 μg), Imp (10 μg), Net (30 μg), Ak (30 μg), CFM (5 μg), Azi (15 μg), Pen G (10 μg), E (15 μg), C (30 μg), Van (30 μg) and Tet (30 μg). All the antibiotic disks were obtained from Oxoid, UK. *E. coli* ATCC 25922 and *Pseudomonas aeruginosa* ATCC 27853 were used as quality control strains.

We were not able to find any standard definition of multidrug-resistance (MDR) and observed that many previous studies have used the definition of MDR as resistance against three or more classes of antibiotics both for Gram-positive [15–17] and Gram-negative [18–21] bacteria. Consequently, the definition of MDR as acquired non-susceptibility against at least three classes of antibiotics was adopted. *Salmonella* species were tested against six classes of antibiotics; penicillin and cephalosporin (AMP, CRO, CFM), aminoglycosides (CN), fluor/quinolones (Cip, NA), sulfonamides (SXT), macrolides (Azi) and chloramphenicol. Other Gram-negative bacteria were tested against seven classes of antibiotics; penicillin and cephalosporin (AMP, CRO, CFM, Caz), carbapenems (Imp, Mem), aminoglycosides (CN, Net, Ak), fluoro/quinolones (Cip, NA), sulfonamides (SXT), macrolides (Azi) and chloramphenicol. Gram-positive bacteria were tested against seven classes of antibiotics; penicillin and cephalosporin (AMP, Pen G, Oxa, CRO, CFM), carbapenems (Imp), aminoglycosides (CN), fluor/quinolones (Cip), sulfonamides (SXT), macrolides (Azi, E) and others (C, Van, Rif).

### Statistical analysis

Blood-borne pathogen trends for isolation and antimicrobial susceptibility over the last ten years was determined using χ^2^ (chi-square) test for trend in SPSS version 16.0. *P* value ≤ 0.05 was considered significant. Standard error of the mean (SEM) was calculated for mean isolation rates. Odd ratios for age and sex were calculated to show their association with infection by particular organisms, and SPSS version 16.0 was used for this purpose. For age, an odds ratio greater than 1.0 indicated the association of a pathogen with age group of less than five years old, and an odds ratio of less than 1.0 indicated the association of a pathogen with age group of more than five years old. For sex, an odds ratio of less than 1.0 indicated the association of a pathogen with female patient group and an odds ratio of more than 1.0 indicated an association of that pathogen with male patient group.

## Results

From January 2005 to December 2014, a total of 103,679 blood samples were received from both hospitalized and domiciliary patients and among them 14015 samples were found to be culture positive. Over these past ten years 11.9, 12.2, 13.9, 16.5, 16.0, 17.3, 12.7, 12.9, 10.7 and 11.9% of the samples were found to be culture positive (*P* < 0.001) respectively. The mean culture positive rate was 13.6 ± 0.7%. Table 1 shows the distribution of organisms found throughout this study period. Blood samples were received from patients with age range of 1 day to 115 years and the mean age was 18 years. *S.* Typhi was the most frequently isolated blood-borne bacterial pathogen in this study, accounting for 36.9% of the total isolates. Other frequently isolated pathogens included coagulase-negative *Staphylococcus* species (21.5%), *Pseudomonas* species (12.5%), *S.* Paratyphi A, B (8.9%) and *Acinetobacter* species (5.1%).

**Table 1:** Bacterial pathogens isolated from blood cultures in Dhaka, Bangladesh from 2005 to 2014

Organisms	Year										Total (14015)
	2005 (1126)	2006 (1132)	2007 (1266)	2008 (1566)	2009 (1522)	2010 (2003)	2011 (1418)	2012 (1391)	2013 (1077)	2014 (1514)	
Salmonella	**566** ^**a**^ **(50.3)** ^**b**^	**747 (66.0)**	**707 (55.8)**	**755 (48.2)**	**593 (39.0)**	**828 (41.3)**	**592 (41.7)**	**663 (47.7)**	**474 (44.0)**	**623 (41.1)**	**6548 (46.7)**
*Salmonella* Typhi	457 (40.6)	595 (52.6)	571 (45.1)	600 (38.3)	483 (31.7)	643 (32.1)	451 (31.8)	527 (37.9)	355 (33.0)	509 (33.6)	5191 (37.0)
*Salmonella* Paratyphi A, B	85 (7.5)	133 (11.7)	127 (10.0)	146 (9.3)	102 (6.7)	179 (8.9)	135 (9.5)	129 (9.3)	107 (9.9)	110 (7.3)	1253 (8.9)
Non-typhoidal *Salmonella* species	24 (2.1)	19 (1.7)	9 (0.7)	9 (0.6)	8 (0.5)	6 (0.3)	6 (0.4)	7 (0.5)	12 (1.1)	4 (0.3)	104 (0.7)
Non-fermenter	**178 (15.8)**	**196 (17.3)**	**232 (18.3)**	**290 (18.5)**	**276 (18.1)**	**338 (16.9)**	**248 (17.5)**	**232 (16.7)**	**187 (17.4)**	**307 (20.3)**	**2484 (17.7)**
*Acinetobacter* species	98 (8.7)	45 (4.0)	101 (8.0)	86 (5.5)	82 (5.4)	68 (3.4)	55 (3.9)	55 (4.0)	56 (5.2)	76 (5.0)	722 (5.2)
*Pseudomonas* species	80 (7.1)	151 (13.3)	131 (10.3)	204 (13.0)	194 (12.7)	270 (13.5)	193 (13.6)	177 (12.7)	131 (12.2)	231 (15.3)	1762 (12.6)
Gram Negative	**146 (13.0)**	**66 (5.8)**	**78 (6.2)**	**85 (5.4)**	**130 (8.5)**	**138 (6.9)**	**114 (8.0)**	**128 (9.2)**	**74 (6.9)**	**116 (7.7)**	**1075 (7.7)**
*Escherichia coli*	35 (3.1)	29 (2.6)	34 (2.7)	46 (2.9)	51 (3.4)	64 (3.2)	48 (3.4)	45 (3.2)	39 (3.6)	36 (2.4)	427 (3.0)
*Klebsiella* species	33 (2.9)	27 (2.4)	31 (2.4)	27 (1.7)	37 (2.4)	51 (2.5)	35 (2.5)	46 (3.3)	29 (2.7)	59 (3.9)	375 (2.7)
*Enterobacter* species	30 (2.7)	8 (0.7)	11 (0.9)	8 (0.5)	42 (2.8)	17 (0.8)	24 (1.7)	27 (1.9)	4 (0.4)	17 (1.1)	188 (1.3)
*Serratia* species	48 (4.3)	2 (0.2)	2 (0.2)	4 (0.3)	0 (0.0)	6 (0.3)	7 (0.5)	10 (0.7)	2 (0.2)	4 (0.3)	85 (0.6)
Gram Positive	**236 (21.0)**	**123 (10.9)**	**249 (19.7)**	**436 (27.8)**	**523 (34.4)**	**699 (34.9)**	**464 (32.7)**	**368 (26.5)**	**342 (31.8)**	**468 (30.9)**	**3908 (27.9)**
Coagulase-negative *Staphylococci*	147 (13.1)	54 (4.8)	179 (14.1)	350 (22.2)	416 (27.3)	591 (29.5)	364 (25.7)	271 (19.5)	248 (23.0)	372 (24.6)	2992 (21.3)
*Staphylococcus aureus*	14 (1.2)	10 (0.9)	15 (1.2)	23 (1.5)	26 (1.7)	30 (1.5)	25 (1.8)	23 (1.7)	23 (2.1)	30 (2.0)	219 (1.6)
*Streptococcus pneumoniae*	49 (4.4)	32 (2.8)	23 (1.8)	23 (1.5)	25 (1.6)	25 (1.2)	29 (2.0)	23 (1.7)	21 (1.9)	26 (1.7)	276 (2.0)
*Streptococcus* species	21 (1.9)	22 (1.9)	17 (1.3)	23 (1.5)	30 (2.0)	27 (1.3)	29 (2.0)	29 (2.1)	35 (3.2)	23 (1.5)	256 (1.8)
*Enterococcus faecalis*	5 (0.4)	5 (0.4)	15 (1.2)	17 (1.1)	26 (1.7)	26 (1.3)	17 (1.2)	22 (1.6)	15 (1.4)	17 (1.1)	165 (1.2)

^a^Values in parentheses indicate the number of isolates found in each year; ^b^Values in parentheses indicate the percentage of isolates found in each year

The bolded data indicates the total number and percentage of that group

*Pseudomonas* species and *Acinetobacter* species were the two major non-fermenter bacteria isolated between 2005 and 2014. *Pseudomonas* species showed a sharp increase in isolation rate, from 40.6 to 79.2% (χ^2^ = 105.1, *P* < 0.001) of the total non-fermenter bacteria, between 2005 and 2010. However, for the next four years their isolation rate decreased slightly and reached 74.3% in 2014. *Acinetobacter* species showed a decreasing trend in their isolation rate over this study period, from 49.8 to 24.4% (χ^2^ = 95.5, *P* < 0.001). *Salmonella* species accounted for 46.7% of the total blood-borne pathogens. We observed an overall decreasing trend in their isolation rate from 2005 to 2014 (χ^2^ = 306.5, *P* < 0.001). Besides non-fermenter and *Salmonella* species, other Gram-negative bacteria constituted only 7.7% (χ^2^ = 72.0, *P* < 0.001) of the total blood-borne pathogens. *E. coli*, *Enterobacter* species and *Serratia* species had a steady isolation rate over the ten year period, while *Klebsiella* species showed an increasing trend in their isolation rate, from 22.6 to 50.9% (χ^2^ = 29.1, *P* < 0.01) of the total Gram-negative bacterial group (excluding *Salmonella* species). Gram-positive bacteria accounted for 27.7% of the total blood-borne pathogens. Their isolation rate increased from 20.6 to 30.8% (χ^2^ = 351.7, *P* < 0.001) over study period. From 2005 to 2014, *Streptococcus pneumoniae* showed a decreasing trend in their isolation rate, from 20.8 to 5.6% (χ^2^ = 158.7, *P* < 0.001) of the total Gram-positive bacteria.

**Fig. 1:**
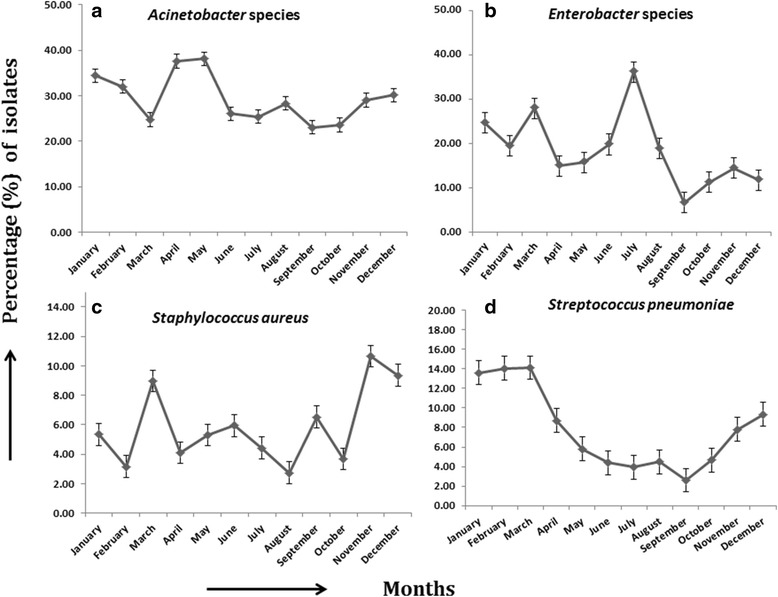
Seasonal variability of BSI causing pathogens- **a** *Acinetobacter* species, **b** *Enterobacter* species, **c** *Staphylococcus aureus*, **d** *Streptococcus pneumoniae*

We observed significant associations between different age groups and infection from specific pathogenic organisms (Additional file 1: Table S7). *Pseudomonas* species (OR: 0.383, 0.341- 0.430, *P* < 0.001), *S.* Paratyphi A, B (0.510, 0.449-0.579, *P* < 0.001) and *Serratia* species (0.592, 0.373-0.941, *P* < 0.05) had significant associations with age group of more than five years old. On the other hand, non-typhoidal *Salmonella* species (5.082, 3.171-8.146, *P* < 0.001) and *S. pneumoniae* (3.827, 2.922-5.012, *P* < 0.001) had significant association with age group of less than five years old. We also observed the association of sex with infection from different pathogenic bacteria (Additional file 2: Table S8). *S. aureus* (1.348, 1.019-1.783, *P* < 0.05) infection was significantly associated with the male patient group, while *E. coli* (0.705, 0.581-0.855, *P* < 0.001) was more associated with the female patient group. Seasonal fluctuation was also observed in the incidence rate of a few pathogens (Fig. 1). *Acinetobacter* species (April-May), *Enterobacter* species (July), *S. aureus* (March and November) and *S. pneumoniae* (March) showed distinct seasonal peaks.

*Acinetobacter* species showed an increasing trend of resistance against gentamicin (χ^2^ = 101.6, *P* < 0.001), ceftriaxone (χ^2^ = 51.8, *P* < 0.001) and ciprofloxacin (χ^2^ = 135.2, *P* < 0.001) (Additional file 3: Table S1). *Pseudomonas* species also showed an increasing trend of resistance against gentamicin (χ^2^ = 127.5, *P* < 0.001), and ciprofloxacin (χ^2^ = 141.7, *P* < 0.001) (Additional file 4: Table S2). Additionally, *Acinetobacter* species and *Pseudomonas* species showed an overall 56.2, 47.6, 32.8, 51.4 and 23.0, 17.4, 56.2, 50.8% resistance against ceftazidime, imipenem, netilmicin and amikacin respectively. Table 2 shows the distribution of their MDR strains over this study period.

**Table 2:** Percentage of MDR strains isolated from blood cultures in Dhaka, Bangladesh from 2005 to 2014

Organisms	Year									
	2005	2006	2007	2008	2009	2010	2011	2012	2013	2014
Salmonella	**286** ^**a**^ **(50.5)** ^**b**^	**383 (51.3)**	**308 (43.6)**	**269 (35.7)**	**189 (31.9)**	**219 (26.4)**	**131 (22.1)**	**109 (16.4)**	**87 (18.4)**	**120 (19.3)**
*Salmonella* Typhi	282 (61.7)	379 (63.7)	307 (53.8)	268 (44.7)	188 (38.9)	219 (34.1)	130 (28.8)	109 (20.7)	87 (24.5)	120 (23.6)
*Salmonella* Paratyphi A, B	1 (1.2)	0 (0.0)	0 (0.0)	0 (0.0)	0 (0.0)	0 (0.0)	1 (0.7)	0 (0.0)	0 (0.0)	0 (0.0)
Non-typhoidal *Salmonella* species	3 (12.5)	4 (21.1)	1 (11.1)	1 (12.5)	1 (12.5)	0 (0.0)	0 (0.0)	0 (0.0)	0 (0.0)	0 (0.0)
Non-Fermenter	**67 (37.6)**	**81 (41.3)**	**85 (36.6)**	**138 (47.6)**	**112 (40.6)**	**101 (29.9)**	**52 (21.0)**	**51 (22.0)**	**57 (30.5)**	**99 (32.2)**
*Acinetobacter* species	24 (24.5)	17 (37.8)	25 (24.8)	37 (43.0)	42 (51.2)	35 (51.5)	23 (41.8)	30 (54.5)	34 (60.7)	50 (65.8)
*Pseudomonas* species	43 (53.8)	64 (42.4)	60 (45.8)	101 (49.5)	70 (36.1)	66 (24.4)	29 (15.0)	21 (11.9)	23 (17.6)	49 (21.2)
Gram-Negative	53 (36.3)	40 (60.6)	45 (57.7)	47 (55.3)	79 (60.8)	98 (71.0)	57 (50.0)	76 (59.4)	53 (71.6)	86 (74.1)
*Escherichia coli*	**22 (62.9)**	**19 (65.5)**	**19 (55.9)**	**23 (50.0)**	**37 (72.5)**	**38 (59.4)**	**25 (52.1)**	**31 (68.9)**	**27 (69.2)**	**26 (72.2)**
*Klebsiella* species	24 (72.7)	18 (66.7)	16 (51.6)	20 (74.1)	21 (56.8)	43 (84.3)	19 (54.3)	24 (52.2)	22 (75.9)	48 (81.4)
*Enterobacter* species	6 (20.0)	3 (37.5)	8 (72.7)	1 (12.5)	21 (50.0)	12 (70.6)	9 (37.5)	12 (44.4)	3 (75.0)	12 (70.6)
*Serratia* species	1 (2.1)	0 (0.0)	2 (100)	3 (75.0)	-	5 (84.3)	4 (57.1)	9 (90.0)	1 (50.0)	0 (0.0)
Gram-Positive	**16 (18.0)**	**16 (23.2)**	**26 (37.1)**	**37 (43.0)**	**51 (47.7)**	**36 (33.3)**	**30 (30.0)**	**27 (27.8)**	**27 (28.7)**	**34 (35.4)**
*Staphylococcus aureus*	4 (28.6)	2 (20.0)	5 (33.3)	16 (69.6)	15 (57.7)	16 (53.3)	14 (56.0)	12 (52.2)	10 (43.5)	19 (63.3)
*Streptococcus pneumoniae*	3 (6.1)	3 (9.4)	3 (13.0)	1 (4.3)	4 (16.0)	4 (16.0)	2 (6.9)	3 (13.0)	6 (28.6)	4 (15.4)
*Streptococcus* species	6 (28.6)	10 (45.5)	6 (35.3)	8 (34.8)	13 (43.3)	10 (37.0)	8 (27.6)	9 (31.0)	9 (25.7)	7 (30.4)
*Enterococcus faecalis*	3 (60)	1 (20.0)	12 (80.0)	12 (70.6)	19 (73.1)	6 (23.1)	6 (35.3)	3 (13.6)	2 (13.3)	4 (23.5)

^a^Indicates the number of MDR strain isolated at that particular year; ^b^indicates the percentage of MDR strains of that particular strain

The bolded data indicates the total number and percentage of that group

*S.* Typhi was found to be consistently sensitive to ceftriaxone and cefixime over this study period; however, a strain of ESBL *S*. Typhi was reported [22]. At the same time, decreasing trends of resistance against ampicillin (χ^2^ = 540.4, *P* < 0.001) and co-trimoxazole (χ^2^ = 740.3, *P* < 0.001) were observed. *S.* Paratyphi A, B was found to be consistently susceptible to ampicillin, co-trimoxazole, ceftriaxone and cefixime over the ten year period. Noticeably, both *S*. Typhi and *S.* Paratyphi A, B showed a trend of increasingly reduced susceptibility against ciprofloxacin (χ^2^ = 24.3 and 66.1 respectively, *P* < 0.001) throughout this study period (Table 3).

**Table 3:** Percentage of antimicrobial resistance in *Salmonella* Typhi and *Salmonella* Paratyphi A, B strains isolated from blood cultures

	*Salmonella* Typhi											*Salmonella* Paratyphi A, B									
	2005	2006	2007	2008	2009	2010	2011	2012	2013	2014		2005	2006	2007	2008	2009	2010	2011	2012	2013	2014
	(457)^a^	(595)	(571)	(600)	(483)	(643)	(451)	(527)	(354)	(509)		(85)^a^	(133)	(127)	(146)	(102)	(179)	(135)	(129)	(107)	(110)
AMP	61	62	54	50	41	41	42	24	28	26	AMP	1	1	0	1	0	0	1	0	1	0
SXT	61	63	54	44	39	34	29	21	24	24	SXT	1	0	0	0	0	0	0	0	1	2
Cip^R^	3	4	2	1	0	2	2	1	1	4	Cip^R^	1	0	0	0	0	0	0	0	0	1
Cip^I^	87	89	88	93	94	94	92	96	92	94	Cip^I^	91	93	97	99	100	99	98	100	98	99
CRO	0	0	0	0	0	0	0	0	0	0	CRO	0	0	0	0	0	0	0	0	0	0
CFM	0	0	0	0	0	0	0	0	1	0	CFM	0	0	1	0	0	0	1	0	0	0

*AMP* ampicillin, *SXT* cotrimoxazole, *Cip* ciprofloxacin, *CRO* ceftriaxone, *CFM* cefixime, ^a^Values in parentheses indicate the number of isolates tested each year

*E. coli* and *Enterobacter* species showed an increasing trend of resistance against gentamicin (χ^2^ = 52.44 and statistically insignificant respectively, *P* < 0.001) and ceftriaxone (χ^2^ = 52.4 and 16.5 respectively, *P* < 0.001). A reducing trend of susceptibility against ciprofloxacin (χ^2^ = 89.9 and 86.8 respectively, *P* < 0.001) was also observed (Table 4 and Additional file 5: Table S4). Additionally, *E. coli* showed an overall increase in resistance, from 69.0 to 90.0%, against co-trimoxazole and ampicillin between 2005 and 2014. *Klebsiella* species showed an increasing trend of resistance against imipenem (χ^2^ = 79.6, *P* < 0.001), and a reducing trend of susceptibility against ciprofloxacin (χ^2^ = 36.1, *P* < 0.001) (Additional file 6: Table S3). Over the same time period an overall increase in resistance, from 62.0 to 76.0%, was observed against gentamicin and ceftriaxone respectively for *Klebsiella species*. Additionally, *Klebsiella species* showed an overall 86.0, 33.0 and 70.0% resistance against cefixime, meropenem and azithromycin respectively from 2010 to 2014.

**Table 4:** Percentage of antimicrobial resistance in *E. coli* strains isolated from blood cultures

	*E. coli*															
	2005	2006	2007	2008	2009	2010	2011	2012	2013	2014		2010	2011	2012	2013	2014
	(35)^a^	(29)	(34)	(46)	(51)	(64)	(48)	(45)	(39)	(36)		(64)	(48)	(45)	(39)	(36)
Amp	88	96	71	79	97	96	93	94	92	92	Net	8	7	7	8	14
SXT	74	75	76	61	73	70	68	73	56	67	Ak	7	4	4	8	14
CN	15	28	24	33	37	36	38	30	44	36	Imp	3	2	4	8	11
Cip^R^	51	48	41	54	63	73	49	71	72	72	Caz	35	30	38	36	56
Cip^I^	0	0	6	2	6	2	2	4	0	0	CFM	69	70	79	68	78
CRO	34	55	32	46	55	63	66	76	69	75						

*AMP* ampicillin, *SXT* cotrimoxazole, *CN* gentamicin, *Cip* ciprofloxacin, *CRO* ceftriaxone, *Net* netilmicin, *Ak* amikacin, *Imp* imipenem, *Caz* ceftazidime, *CFM* cefixime, ^a^Values in parentheses indicate the number of isolates tested each year

*S. pneumoniae* was found to be consistently susceptible to ampicillin and ceftriaxone between 2005 and 2014 (Table 5). Over this period, their resistance to co-trimoxazole reduced (χ^2^ = 92.5, *P* < 0.001). However, it increased against penicillin G (χ^2^ = 195.3, *P* < 0.001) and erythromycin (χ^2^ = 293.2, *P* < 0.001). Also, a trend of increased susceptibility was observed against ciprofloxacin (χ^2^ = 79.2, *P* < 0.001). Other *Streptococcus* species showed an increasing trend of resistance against gentamicin (χ^2^ = 44.9, *P* < 0.001) and erythromycin (χ^2^ = 70.0, *P* < 0.001), while there was a reducing trend in susceptibility against ciprofloxacin (χ^2^ = 67.3, *P* < 0.001) (Additional file 7: Table S5). *S. aureus* was found to be highly resistant to ampicillin (up to 100%) throughout this study (Table 6). For this species, resistance against erythromycin increased from 36.0% in 2005 to 75.0% in 2014 (*P* < 0.09) while susceptibility to ciprofloxacin reduced (χ^2^ = 37.2, *P* < 0.001). *S. aureus* remained consistently susceptible to vancomycin for the last five years of the study and during the same time period they showed an overall 42.0% resistance against ceftriaxone. *E. faecalis* was found consistently susceptible to vancomycin between 2010 and 2014, while this species showed an overall 31.0, 45.0, 60.0, 39.0 and 81.0% resistance against ampicillin, gentamicin (120), ciprofloxacin, penicillin G and co-trimoxazole respectively (Additional file 8: Table S6).

**Table 5:** Percentage of antimicrobial resistance in *Streptococcus pneumoniae* strains isolated from blood cultures

	*Streptococcus pneumoniae*									
	2005	2006	2007	2008	2009	2010	2011	2012	2013	2014
	(49)^a^	(32)	(23)	(23)	(25)	(25)	(29)	(23)	(21)	(26)
Amp	2	0	0	0	0	0	0	0	0	0
SXT	91	90	87	73	76	91	78	70	71	73
CipR	6	0	5	0	4	0	8	0	0	0
CipI	22	38	24	5	16	0	8	0	9	9
CRO	0	0	0	0	0	0	0	0	0	0
Pen G	2	0	0	0	9	8	3	4	5	8
E	4	0	0	0	8	5	24	23	48	31
Azi	-	-	-	-	-	7	39	14	55	31
CFM	-	-	-	-	-	17	0	7	0	8

*AMP* ampicillin, *SXT* cotrimoxazole, *Cip* ciprofloxacin, *CRO* ceftriaxone, *Pen G* penicillin G, *E* erythromycin, *Azi* azithromycin, *CFM* cefixime. ^a^Values in parentheses indicate the number of isolates tested each year; − indicates absence of data in respective years

**Table 6:** Percentage of antimicrobial resistance in *Staphylococcus aureus* strains isolated from blood cultures

	*Staphylococcus aureus*									
	2005	2006	2007	2008	2009	2010	2011	2012	2013	2014
	(14)^a^	(10)	(15)	(23)	(26)	(30)	(25)	(23)	(23)	(30)
Amp	100	100	88	90	88	67	100	100	100	71
SXT	29	20	20	52	35	33	36	23	17	38
C	7	0	7	4	4	4	0	13	4	17
E	36	20	33	78	81	67	72	73	64	75
CipR	25	20	40	65	54	57	64	48	39	67
CipI	8	0	0	0	4	3	4	9	13	0
CN	25	11	20	26	27	33	24	13	22	27
CRO	-	-	-	-	-	43	29	50	41	45
Van	-	-	-	-	-	4	0	0	0	0

*AMP* ampicillin, *SXT* cotrimoxazole, *C* chloramphenicol, *E* erythromycin, *Cip* ciprofloxacin, *CN* gentamicin, *CRO* ceftriaxone, *Van* vancomycin, ^a^Values in parentheses indicate the number of isolates tested each year; − indicates absence of data in respective years

## Discussion

In this retrospective study, we aimed to identify the most prevalent pathogenic organisms involved in bloodstream infections (BSI) over a ten year period (2005–14) in patients in Dhaka, Bangladesh. We also aimed to determine the antimicrobial susceptibility of the isolated pathogens against multiple antibiotics to achieve a clear outlook on the changing trend of their antibiotic susceptibility.

*S*. Typhi, the causative agent of typhoid fever, is a major public health concern in Bangladesh and other developing Asian countries. Several studies from Bangladesh have already identified *S*. Typhi as a common cause of bloodstream infection in this region [23–25]. We found *Salmonella* species to be responsible for almost half of the disease burden associated with BSI in Dhaka, Bangladesh and about 80% of these infections were due to *S.* Typhi. However, we have observed an overall decrease in *Salmonella* species isolation rate over this study period. This decrease may be attributed to the improved urban water management system and sanitation practices in Dhaka city over the past years.

**Fig. 2:**
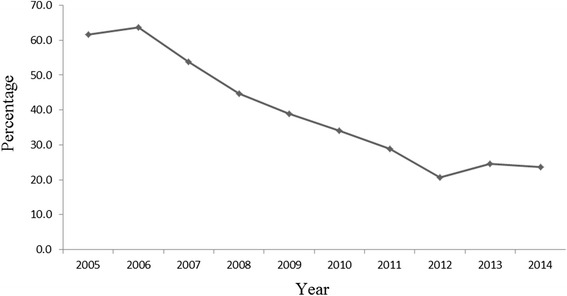
Decrease of multidrug-resistant (MDR) *Salmonella* Typhi isolation rate from blood cultures

A significant change in the epidemiology of *S.* Typhi was observed in early 1990’s as those years experienced a dramatic rise of MDR strains in Dhaka, Bangladesh [26]. By mid-1990s, about half of the *S.* Typhi strains were MDR; these were resistant against three first line antibiotics - ampicillin, cotrimoxazole and chloramphenicol [26]. Noticeably, after a few years of an initial epidemic period, a decreasing trend in the isolation rate of MDR *S.* Typhi strains was observed [23]. Our study confirms this trend to continue; from 2005 to 2010, the percentage of MDR *S.* Typhi strains isolated declined from 61.7 to 23.7% (Fig. 2). As indicated by recent studies from Bangladesh, *S.* Typhi still shows a high level of resistance against first line antibiotics [23]. However, we have observed a steady decrease in resistance against ampicillin and cotrimoxazole over the ten year study period. Hopefully, if this trend continues, then using the cheaper, first line antibiotics again to treat *S.* Typhi infections might be a possibility in near future. Ciprofloxacin or ceftriaxone is usually the choice of treatment against MDR *Salmonella* strains [27]. We found all *Salmonella* isolates were consistently susceptible to ceftriaxone, but a very high level of reduced susceptibility exists against ciprofloxacin. Studies suggest that the presence of a mutation in the quinolone resistance-determining region (QRDR) of the gyrA gene is responsible for the emergence of this reduced susceptibility [28].

Non-fermenter Gram-negative bacilli are well known for their ability to cause nosocomial infections [29, 30]. We identified *Pseudomonas* species and *Acinetobacter* species to be the third (12.6%) and fifth (5.2%) most prevalent organisms associated with BSI in Dhaka, Bangladesh. A gradual decrease in the presence of *Acinetobacter* species and a significant increase in the presence of *Pseudomonas* species were observed over the study period. This increase in BSI from *Pseudomonas* species was possibly from the patients increased access to the health care facilities over these years. Also noticeably, *Pseudomonas* species was found to be more associated with patients above five years of age; almost 70% of the infections occurring among adults (≥18 years of age). A recent study from Bangladesh also suggested that there is a very low amount of bacteremia cases due to *Pseudomonas* species among diarrheal children less than five years of age [31].

*Acinetobacter* species and *Pseudomonas* species are well known for their high degree of resistance against all classes of antibiotics, and the emergence of MDR strains makes it very difficult to treat them. An aminoglycosides combined with a β-lactamase-stable β-lactam is the typical choice of treatment for *Acinetobacter* species infection [32], and carbapenems are known to be most effective against their MDR strains [33]. We observed *Acinetobacter* species to develop increasingly high levels of antibiotic resistance against all aminoglycosides, and noticeably, very high levels of resistance against imipenem (64%). Usually, in the case of resistance against carbapenems, the expert’s choice of treatment for *Acinetobacter* species infections entails colistin and tigecycline [34]. We found only 3.73% of the MDR *Acinetobacter* species strains to be resistant against polymyxin B (300 u), therefore it might be considered as a good treatment choice against MDR *Acinetobacter* species. Colistin and polymyxin B (300 u) are usually considered as the last resort for treatment of infections due to MDR *Pseudomonas* species [35], but we observed high levels of resistance against them. In contrast, we found imipenem to be consistently effective against all *Pseudomonas* species so it might be considered as a good choice of treatment.

Besides non-fermenter and *Salmonella* species, other clinically important Gram-negative bacteria were *E. coli*, *Enterobacter* species, *Klebsiella* species and *Serratia* species. *E. coli* has been reported as the leading cause of BSI in developed countries [1]; however, data is relatively unavailable for Bangladesh. We found *E. coli* to be responsible for only 3.05% of the total BSI cases. It is likely that the presence of *E. coli* is overshadowed by the overwhelming presence of *Salmonella* species in Dhaka, Bangladesh, so it would be unwise to ignore them as clinically insignificant.

A recent study from Bangladesh has shown a very high level of antibiotic resistance and MDR level among *E. coli* isolates from environmental samples [36]. In this study we identified a much higher level of antibiotic resistance and MDR level (36% MDR in environment samples vs. 62.38% MDR in our clinical samples) among BSI causing *E. coli* strains. We found carbapenems to be the most active antibiotics against *E. coli*. Overall, 3.2% (including MDR strains) of the *E. coli* strains showed resistance against them. So, carbapenems may be considered as a good choice of treatment for BSI caused by *E. coli. Klebsiella* species showed the highest level of resistance against β-lactams, especially penicillins and third generation cephalosporins. Previous studies suggested that carbapenems were highly effective against *Klebsiella* species until the early years of 2000s. According to Centers for Disease Control and Prevention (CDC) in the USA, *Klebsiella* species acquired the highest amount of carbapenem resistance (from 1.6 to 10.4%) in the decade from 2001–2011. In accordance with this finding, we observed a very dramatic rise in *Klebsiella* species resistance against imipenem (0 to 46%) and meropenem (0 to 46.5%) from 2005 to 2014. In fact, we found *Klebsiella* species to show the highest level of MDR (68%) among all the isolated pathogens. Overall, *E. coli*, *Klebsiella* species and *Enterobacter* species had relatively low prevalence throughout this study period. However, they should be considered important as they had the highest amount of MDR strains and thus the potential to cause serious health threats.

Gram-positive bacteria also pose serious threats as their incidence in BSI is increasing steadily worldwide. The problem is particularly acute in nosocomial settings where methicillin-resistant *S. aureus* (MRSA) and vancomycin resistant *Enterococcus* (VRE) are considered serious health threats [37, 38]. In this study, we observed an increasing trend in the isolation rate of Gram-positive bacteria. This trend may be attributed to the patient’s increased access to the health care facilities where BSI from Gram-positive bacteria is common. We identified *S. aureus* and *S. pneumoniae* as the two major Gram-positive pathogenic bacteria associated with BSI in Dhaka, Bangladesh. *S. pneumoniae* reportedly kills about 0.7 to 1 million children under five years of age every year worldwide and 90% of these deaths occur in developing countries [39]. Pneumonia is the primary cause of childhood death in Bangladesh [40] and studies suggest that the incidence of pneumonia among preschool children in Bangladesh is surprisingly higher than in developed countries [41, 42]. We found almost 75% of the BSI cases from *S. pneumoniae* to occur among children less than five years of age. However, a decreasing trend in their isolation rate was observed throughout this study period.

Increasing levels of antibiotic resistance are a big concern for *S. pneumoniae*, especially their resistance against macrolides and β-lactams, which are the first choice of treatment for most pneumonia cases. One recent review indicates a very high level of penicillin resistance among *S. pneumoniae* strains in different Asian countries [43]. However, we observed only a small increase in penicillin resistance in Dhaka, Bangladesh. Another study from Dhaka indicates the presence of high level of resistance against ciprofloxacin. However our study found a much lower level of resistance against ciprofloxacin and also we observed a decreasing trend in their susceptibility pattern [44]. Ampicillin and ceftriaxone were found to be consistently active against *S. pneumoniae*, so they might be considered as good treatment choices for them. Emergence of MRSA strain marks another very important epidemiological change for the past few decades [45]. Naturally, *S. aureus* are susceptible to commonly used antibiotics. However methicillin resistance is associated with resistance against a broad range of antibiotics [46]. Over the study period, we found overall 50.2% of *S. aureus* strains to be MDR. In the first five years of our study, we identified 34.4% of the *S. aureus* strains to be oxacillin (a synthetic form of methicillin) resistant; however, the percentage was much higher (58.7%) amongst the MDR strains. Vancomycin has been widely used as the preferred choice of treatment for MRSA strains [47], and we also found vancomycin to be highly effective against all *S. aureus* strains; only 2.1% strains were resistant. Linezolid was also found to be very effective against *S. aureus*; only one resistant strain was detected over the study period. Therefore, both vancomycin and linezolid might be considered as a good treatment choices against *S. aureus* which are resistant to commonly used antibiotics.

### Limitations of the study

Due to the lack of resources, we were not able to differentiate the samples received from our hospital patients and from domiciliary patients. As a result we could not show the difference in epidemiology between nosocomial and community acquired BSI. Also due to lack of resources, we were not able to collect patient data on the clinical manifestations or any other patient characteristics, other than age and sex, which could be considered as risk factors for BSI. However, we confirm that each patient was diagnosed with signs of bacteremia by their respective physicians. Also, we were not able to perform any molecular tests on received samples due to lack of required resources.

## Conclusion

This study clearly identifies the bacterial pathogens involved with bloodstream infections (BSI) occurring in Dhaka, Bangladesh between January 2005 to December 2014. It also reveals their antibiotic susceptibility patterns for commonly used antibiotics. We expect our findings to help healthcare professionals to make informed decisions and provide better care for their patients. Also, we hope this study to help researchers and policy makers to prioritize their research options to face future challenges of infectious diseases both at home and abroad.

## Additional files

Additional file 1: Table S7. Association of two age groups with distinct bacterial pathogens causing BSI in Dhaka, Bangladesh. (DOC 38 kb)
Additional file 2: Table S8. Association of sex with distinct bacterial pathogens causing BSI in Dhaka, Bangladesh. (DOC 40 kb)
Additional file 3: Table S1. Percentage of antimicrobial resistance in *Acinetobacter* species strains isolated from blood cultures. (DOC 35 kb)
Additional file 4: Table S2. Percentage of antimicrobial resistance in *Pseudomonas* species strains isolated from blood cultures. (DOC 35 kb)
Additional file 5: Table S4. Percentage of antimicrobial resistance in *Enterobacter* species strains isolated from blood samples. (DOC 33 kb)
Additional file 6: Table S3. Percentage of antimicrobial resistance in *Klebsiella* species strains isolated from blood cultures. (DOC 38 kb)
Additional file 7: Table S5. Percentage of antimicrobial resistance in *Streptococcus* species strains isolated from blood cultures. (DOC 36 kb)
Additional file 8: Table S6. Percentage of antimicrobial resistance in *Enterococcus faecalis* strains isolated from blood cultures. (DOC 36 kb)

## Abbreviations

FAN: Fastidious antibiotic neutralization
API: Analytical profile index
