# Supplementary material for: Bacterial etiology of bloodstream infections and antimicrobial resistance in Dhaka, Bangladesh, 2005–2014
Source: Antimicrob Resist Infect Control. 2017 Jan 5;6:2. doi: 10.1186/s13756-016-0162-z (PMC5217397; doi:10.1186/s13756-016-0162-z)
Supplement: Additional file 1: Table S7. — Association of two age groups with distinct bacterial pathogens causing BSI in Dhaka, Bangladesh. (DOC 38 kb) [file 13756_2016_162_MOESM1_ESM.doc]

**Additional file 1 Table S7:** Association of two age groups with distinct bacterial pathogens causing BSI in

Dhaka, Bangladesh.

| Organism | Age < 5 y  (n= 4146)^a^ | Age > 5 y  (n = 6843)^a^ | Odd Ratio (95% CI) | P Value |
| --- | --- | --- | --- | --- |
| *Acinetobacter* species | 326 (7.9) | 393 (5.7) | 1.125 (0.968-1.309) | 0.125 |
| *Pseudomonas* species | 420 (10.1) | 1328 (19.4) | 0.383 (0.341-0.430) | <0.001 |
|  |  |  |  |  |
| Non-typhoidal *Salmonella* species | 82 (2.0) | 22 (0.3) | 5.082 (3.171-8.146) | <0.001 |
| *Salmonella* Typhi | 2068 (49.9) | 3117 (45.6) | 0.839 (0.783-0.899 ) | <0.001 |
| *Salmonella* Paratyphi A, B | 358 (8.6) | 895 (13.1) | 0.510 (0.449-0.579) | <0.001 |
|  |  |  |  |  |
| *Enterococcus faecalis* | 58 (1.4) | 106 (1.5) | 0.735 (0.533-1.014) | 0.060 |
| *Staphylococcus aureus* | 82 (2.0) | 137 (2.0) | 0.804 (0.611-1.060) | 0.121 |
| *Streptococcus pneumoniae* | 202 (4.9) | 73 (1.1) | 3.827 (2.922-5.012) | <0.001 |
| *Streptococcus* species | 129 (3.1) | 127 (1.9) | 1.378 (1.076-1.764) | <0.05 |
|  |  |  |  |  |
| *Enterobacter* species | 65 (1.6) | 121 (1.8) | 0.721 (0.533-0.977) | <0.05 |
| *Escherichia coli* | 178 (4.3) | 249 (3.6) | 0.963 (0.792-1.171) | 0.704 |
| *Klebsiella* species | 152 (3.7) | 216 (3.2) | 0.948 (0.768-1.169) | 0.616 |
| *Serratia* species | 26 (0.6) | 59 (0.9) | 0.592 (0.373-0.941) | <0.05 |

CI: Confidence interval, ^a^Values presented here are as No. (%)
