# Supplementary material for: Bacterial etiology of bloodstream infections and antimicrobial resistance in Dhaka, Bangladesh, 2005–2014
Source: Antimicrob Resist Infect Control. 2017 Jan 5;6:2. doi: 10.1186/s13756-016-0162-z (PMC5217397; doi:10.1186/s13756-016-0162-z)
Supplement: Additional file 2: Table S8. — Association of sex with distinct bacterial pathogens causing BSI in Dhaka, Bangladesh. (DOC 40 kb) [file 13756_2016_162_MOESM2_ESM.doc]

**Additional file 2 Table S8**: Association of sex with distinct bacterial pathogens causing BSI in Dhaka, Bangladesh.

| Organism | Male  (n = 6316)^a^ | Female  (n = 4696)^a^ | Odd Ratio (95% CI) | P Value |
| --- | --- | --- | --- | --- |
| *Acinetobacter* species | 442 (7.0) | 278 (5.9) | 1.175 (1.007-1.370) | <0.05 |
| *Pseudomonas* species | 1065 (16.9) | 696 (14.8) | 1.140 (1.029-1.262) | <0.05 |
|  |  |  |  |  |
| Non-typhoidal *Salmonella* species | 64 (1.0) | 40 (0.9) | 1.174 (0.790-1.745) | 0.427 |
| *Salmonella* Typhi | 2898 (45.9) | 2290 (48.8) | 0.887 (0.828-0.951) | <0.01 |
| *Salmonella* Paratyphi A, B | 716 (11.3) | 536 (11.4) | 0.977 (0.869-1.011) | 0.699 |
|  |  |  |  |  |
| *Enterococcus faecalis* | 97 (1.5) | 66 (1.4) | 1.078 (0.787-1.477) | 0.639 |
| *Staphylococcus aureus* | 141 (2.2) | 77 (1.6) | 1.348 (1.019-1.783) | <0.05 |
| *Streptococcus pneumoniae* | 160 (2.5) | 116 (2.5) | 1.011 (0.794-1.287) | 0.928 |
| *Streptococcus* species | 151 (2.4) | 105 (2.2) | 1.055 (0.821-1.357) | 0.676 |
|  |  |  |  |  |
| *Enterobacter* species | 103 (1.6) | 85 (1.8) | 0.887 (0.664-1.184) | <0.01 |
| *Escherichia coli* | 210 (3.3) | 216 (4.6) | 0.705 (0.581-0.855) | <0.001 |
| *Klebsiella* species | 232 (3.7) | 143 (3.0) | 1.195 (0.967-1.476) | 0.098 |
| *Serratia* species | 37 (0.6) | 48 (1.0) | 0.563 (0.366-0.866) | <0.01 |

CI: Confidence interval; ^a^Values presented here are as No. (%)
