# Supplementary material for: Bacterial etiology of bloodstream infections and antimicrobial resistance in Dhaka, Bangladesh, 2005–2014
Source: Antimicrob Resist Infect Control. 2017 Jan 5;6:2. doi: 10.1186/s13756-016-0162-z (PMC5217397; doi:10.1186/s13756-016-0162-z)
Supplement: Additional file 3: Table S1. — Percentage of antimicrobial resistance in Acinetobacter species strains isolated from blood cultures. (DOC 35 kb) [file 13756_2016_162_MOESM3_ESM.doc]

**Additional file 3 Table S1:** Percentage of antimicrobial resistance in *Acinetobacter* species strains isolated from blood cultures.

|  | *Acinetobacter* species | | | | | | | | | | | | | | | |
| --- | --- | --- | --- | --- | --- | --- | --- | --- | --- | --- | --- | --- | --- | --- | --- | --- |
|  | 2005 | 2006 | 2007 | 2008 | 2009 | 2010 | 2011 | 2012 | 2013 | 2014 |  | 2010 | 2011 | 2012 | 2013 | 2014 |
|  | (98)* | (45) | (101) | (86) | (82) | (68) | (55) | (55) | (56) | (76) |  | (68) | (55) | (55) | (56) | (76) |
| CN | 27 | 45 | 32 | 42 | 52 | 64 | 55 | 63 | 63 | 64 | Caz | 64 | 44 | 51 | 55 | 67 |
| Cip^R^ | 27 | 20 | 14 | 36 | 43 | 49 | 44 | 44 | 54 | 48 | Imp | 39 | 35 | 45 | 55 | 64 |
| Cip^I^ | 2 | 9 | 2 | 3 | 4 | 3 | 11 | 7 | 4 | 9 | Net | 38 | 17 | 29 | 41 | 39 |
| CRO | 35 | 41 | 35 | 38 | 44 | 63 | 45 | 52 | 57 | 71 | Ak | 51 | 42 | 51 | 56 | 57 |

CN, gentamicin; Cip, ciprofloxacin; CRO, ceftriaxone; Caz, ceftazidime; Imp, imipenem; Net, netilmicin ; Ak, amikacin.

* Values in parentheses indicate the number of isolates tested each year.
