# Supplementary material for: Bacterial etiology of bloodstream infections and antimicrobial resistance in Dhaka, Bangladesh, 2005–2014
Source: Antimicrob Resist Infect Control. 2017 Jan 5;6:2. doi: 10.1186/s13756-016-0162-z (PMC5217397; doi:10.1186/s13756-016-0162-z)
Supplement: Additional file 4: Table S2. — Percentage of antimicrobial resistance in Pseudomonas species strains isolated from blood cultures. (DOC 35 kb) [file 13756_2016_162_MOESM4_ESM.doc]

**Additional file 4 Table S2:** Percentage of antimicrobial resistance in *Pseudomonas* species strains isolated from blood cultures.

|  | *Pseudomonas* species | | | | | | | | | | | | | | | |
| --- | --- | --- | --- | --- | --- | --- | --- | --- | --- | --- | --- | --- | --- | --- | --- | --- |
|  | 2005 | 2006 | 2007 | 2008 | 2009 | 2010 | 2011 | 2012 | 2013 | 2014 |  | 2010 | 2011 | 2012 | 2013 | 2014 |
|  | (80)* | (151) | (131) | (204) | (194) | (270) | (193) | (177) | (131) | (231) |  | (270) | (193) | (177) | (131) | (231) |
| CN | 47 | 49 | 45 | 75 | 71 | 74 | 79 | 67 | 76 | 84 | CAZ | 28 | 19 | 20 | 25 | 23 |
| Cip^R^ | 14 | 0 | 6 | 8 | 18 | 25 | 15 | 22 | 20 | 21 | Imp | 29 | 16 | 12 | 14 | 16 |
| Cip^I^ | 0 | 2 | 0 | 1 | 1 | 2 | 6 | 4 | 7 | 5 | Net | 51 | 43 | 47 | 63 | 77 |
|  |  |  |  |  |  |  |  |  |  |  | Ak | 41 | 41 | 40 | 60 | 72 |

CN, gentamicin; Cip, ciprofloxacin; Caz, ceftazidime; Imp, imipenem; Net, netilmicin; Ak, amikacin. * Values in parentheses indicate the number of isolates tested each year.
