# Supplementary material for: Bacterial etiology of bloodstream infections and antimicrobial resistance in Dhaka, Bangladesh, 2005–2014
Source: Antimicrob Resist Infect Control. 2017 Jan 5;6:2. doi: 10.1186/s13756-016-0162-z (PMC5217397; doi:10.1186/s13756-016-0162-z)
Supplement: Additional file 5: Table S4. — Percentage of antimicrobial resistance in Enterobacter species strains isolated from blood samples. (DOC 33 kb) [file 13756_2016_162_MOESM5_ESM.doc]

**Additional file 5 Table S4:** Percentage of antimicrobial resistance in *Enterobacter* species strains isolated from blood samples.

|  | *Enterobacter* species | | | | | | | | | |
| --- | --- | --- | --- | --- | --- | --- | --- | --- | --- | --- |
|  | 2005 | 2006 | 2007 | 2008 | 2009 | 2010 | 2011 | 2012 | 2013 | 2014 |
|  | (30) | (8) | (11) | (8) | (42) | (17) | (24) | (27) | (4) | (17) |
| CN | 20 | 38 | 50 | 13 | 69 | 69 | 38 | 52 | 25 | 47 |
| Cip^R^ | 7 | 13 | 64 | 0 | 5 | 29 | 25 | 22 | 0 | 41 |
| Cip^I^ | 3 | 25 | 18 | 0 | 69 | 47 | 4 | 4 | 25 | 24 |
| CRO | 21 | 50 | 60 | 13 | 83 | 80 | 38 | 48 | 50 | 65 |
| Imp | - | - | - | - | - | 6 | 4 | 0 | 25 | 12 |
| CFM | - | - | - | - | - | 100 | 42 | 60 | 75 | 82 |

CN, gentamicin; Cip, ciprofloxacin; CRO, ceftriaxone ; Imp, imipenem; CFM, cefixime; *Values in parentheses indicate the number of isolates tested each year.
