# Supplementary material for: Bacterial etiology of bloodstream infections and antimicrobial resistance in Dhaka, Bangladesh, 2005–2014
Source: Antimicrob Resist Infect Control. 2017 Jan 5;6:2. doi: 10.1186/s13756-016-0162-z (PMC5217397; doi:10.1186/s13756-016-0162-z)
Supplement: Additional file 6: Table S3. — Percentage of antimicrobial resistance in Klebsiella species strains isolated from blood cultures. (DOC 38 kb) [file 13756_2016_162_MOESM6_ESM.doc]

**Additional file 6 Table S3:** Percentage of antimicrobial resistance in *Klebsiella* species strains isolated from blood cultures.

|  | *Klebsiella* species | | | | | | | | | | | | | | | | |
| --- | --- | --- | --- | --- | --- | --- | --- | --- | --- | --- | --- | --- | --- | --- | --- | --- | --- |
|  | 2005 | 2006 | 2007 | 2008 | 2009 | 2010 | 2011 | 2012 | 2013 | 2014 |  | 2010 | 2011 | 2012 | 2013 | 2014 |  |
|  | (33)* | (27) | (31) | (27) | (37) | (51) | (35) | (46) | (29) | (59) |  | (51) | (35) | (46) | (29) | (59) |  |
| CN | 73 | 59 | 55 | 70 | 46 | 84 | 54 | 57 | 59 | 64 | CFM | 92 | 88 | 71 | 89 | 92 |  |
| Cip^R^ | 38 | 44 | 32 | 59 | 51 | 73 | 47 | 46 | 62 | 63 | Mem | 27 | 32 | 28 | 32 | 46 |  |
| Cip^I^ | 25 | 7 | 16 | 11 | 8 | 12 | 24 | 20 | 17 | 20 | Azi^R^ | 75 | 58 | 61 | 75 | 83 |  |
| CRO | 75 | 67 | 55 | 81 | 62 | 90 | 86 | 67 | 86 | 93 | Azi^I^ | 20 | 27 | 24 | 21 | 12 |  |
| Imp | 0 | 0 | 0 | 17 | 9 | 26 | 34 | 28 | 34 | 46 |  |  |  |  |  |  |  |

CN, gentamicin; Cip, ciprofloxacin; CRO, ceftriaxone ; Imp, imipenem; CFM, cefixime; Mem, meropenem; Azi, azithromycin

* Values in parentheses indicate the number of isolates tested each year.
