# Supplementary material for: Bacterial etiology of bloodstream infections and antimicrobial resistance in Dhaka, Bangladesh, 2005–2014
Source: Antimicrob Resist Infect Control. 2017 Jan 5;6:2. doi: 10.1186/s13756-016-0162-z (PMC5217397; doi:10.1186/s13756-016-0162-z)
Supplement: Additional file 7: Table S5. — Percentage of antimicrobial resistance in Streptococcus species strains isolated from blood cultures. (DOC 36 kb) [file 13756_2016_162_MOESM7_ESM.doc]

**Additional file 7 Table S5:** Percentage of antimicrobial resistance in *Streptococcus* species strains isolated from blood cultures.

|  | *Streptococcus* species | | | | | | | | | |
| --- | --- | --- | --- | --- | --- | --- | --- | --- | --- | --- |
|  | 2005 | 2006 | 2007 | 2008 | 2009 | 2010 | 2011 | 2012 | 2013 | 2014 |
|  | (21)* | (22) | (17) | (23) | (30) | (27) | (29) | (29) | (35) | (23) |
| Amp | 0 | 38 | 0 | 14 | 16 | 8 | 19 | 0 | 0 | 14 |
| CN | 33 | 50 | 33 | 25 | 29 | 46 | 52 | 52 | 60 | 50 |
| CRO | 0 | 30 | 18 | 4 | 20 | 7 | 14 | 7 | 6 | 18 |
| Pen G | 5 | 35 | 0 | 14 | 17 | 19 | 14 | 0 | 3 | 18 |
| E | 45 | 44 | 29 | 39 | 52 | 56 | 45 | 45 | 49 | 61 |
| SXT | 85 | 86 | 82 | 83 | 97 | 78 | 60 | 89 | 90 | 75 |
| Cip^R^ | 27 | 53 | 12 | 39 | 57 | 70 | 57 | 33 | 20 | 33 |
| Cip^I^ | 0 | 13 | 18 | 17 | 23 | 10 | 29 | 67 | 20 | 17 |

Amp, ampicillin; CN, gentamicin; CRO, ceftriaxone; Pen G, penicillin G; E, erythromycin; SXT, cotrimoxazole; Cip, ciprofloxacin; *Values in parentheses indicate the number of isolates tested each year.
