# Supplementary material for: Bacterial etiology of bloodstream infections and antimicrobial resistance in Dhaka, Bangladesh, 2005–2014
Source: Antimicrob Resist Infect Control. 2017 Jan 5;6:2. doi: 10.1186/s13756-016-0162-z (PMC5217397; doi:10.1186/s13756-016-0162-z)
Supplement: Additional file 8: Table S6. — Percentage of antimicrobial resistance in Enterococcus faecalis strains isolated from blood cultures. (DOC 36 kb) [file 13756_2016_162_MOESM8_ESM.doc]

**Additional file 8 Table S6:** Percentage of antimicrobial resistance in *Enterococcus faecalis* strains isolated from blood cultures.

|  | *Enterococcus faecalis* | | | | | | | | | |
| --- | --- | --- | --- | --- | --- | --- | --- | --- | --- | --- |
|  | 2005 | 2006 | 2007 | 2008 | 2009 | 2010 | 2011 | 2012 | 2013 | 2014 |
|  | (5)* | (5) | (15) | (17) | (26) | (26) | (17) | (22) | (15) | (17) |
| Amp | 0 | 20 | 38 | 18 | 20 | 42 | 62 | 38 | 20 | 47 |
| CN | 0 | 25 | 29 | 54 | 60 | 71 | 71 | 36 | 36 | 67 |
| Cip^R^ | 25 | 25 | 43 | 65 | 62 | 88 | 94 | 68 | 53 | 77 |
| Cip^I^ | 0 | 25 | 21 | 35 | 31 | 8 | 6 | 14 | 27 | 15 |
| Pen G | 0 | 25 | 50 | 31 | 32 | 52 | 76 | 43 | 27 | 50 |
| SXT | 100 | 100 | 80 | 100 | 100 | 75 | 100 | 0 | 100 | 50 |
| Van | - | - | - | - | - | 8 | 6 | 0 | 0 | 0 |

Amp, ampicillin; CN, Gentamicin; Cip, ciprofloxacin; Pen G, penicillin G; SXT, Cotrimoxazole; Van, vancomycin

* Values in parentheses indicate the number of isolates tested each year.
